# Incidence rate of congenital scoliosis estimated from a nationwide health insurance database

**DOI:** 10.1038/s41598-021-85088-7

**Published:** 2021-03-09

**Authors:** Ji-Won Kwon, Hyun Wook Chae, Hye Sun Lee, Sinae Kim, Sahyun Sung, Soo Bin Lee, Seong-Hwan Moon, Hwan-Mo Lee, Byung Ho Lee

**Affiliations:** 1 Department of Pediatrics, Yonsei University College of Medicine, Seoul, Korea; 2 Biostatistics Collaboration Unit, Yonsei University College of Medicine, Seoul, Korea; 3 Department of Orthopedic Surgery, Yonsei University College of Medicine, 50 Yonsei-ro, Seodaemun-gu, Seoul, 120-752 Korea; 4 Department of Orthopedic Surgery, National Health Insurance Corporation Ilsan Hospital, Goyang, Korea; 5 Department of Orthopedic Surgery, Bundang Jesaeng General Hospital, Daejin Medical Center, Seongnam, Korea; 6 Department of Orthopedic Surgery, Ewha Womans University College of Medicine, Seoul, Korea

**Keywords:** Diseases, Medical research

## Abstract

To investigate the epidemiology of congenital scoliosis (CS) and treatment trends. An age-matched, nationwide, population-based study was conducted using the National Health Insurance and Health Insurance Review and Assessment databases from 2010 to 2015. Data regarding the diagnosis and treatment of scoliosis were extracted using International Classifications of Diseases, 10th revision codes. The age-matched normal population was determined from the Korean Statistical Information Service database. We analyzed the incidence rate of CS according to age and sex, as well as the proportion of surgically treated patients. A total of 1664 patients (aged 0–19 years) were diagnosed with CS. The overall average incidence rate of CS over the 5-year period was 3.08 per 100,000 persons, with the highest and second highest rates at 0 years and 12–16 years of age, respectively. The incidence rate stratified by age ranged from 1.5 to 20.1 per 100,000 persons among the age-matched normal population, with peaks at 0 years of age and the second growth spurt in adolescence (12–16 years for males; 10–14 years for females). Anterior surgery was rarely performed; posterior surgery was performed in 92 patients (5.5% of all patients), with the highest prevalence (56.5%) in patients diagnosed at 10–14 years of age. The overall average incidence rate of CS over a 5-year period was 3.08 per 100,000 persons. Only 5.5% of patients underwent surgery within 5 years after the initial diagnosis.

## Introduction

Congenital scoliosis (CS) is a spinal malformation resulting in longitudinal and rotational imbalances due to heterotopic vertebra and rib bone formation^1^. CS very often has accompanying anomalies, known as VACTERL association-type anomalies^2^. VACTERL association-type anomalies refer to the co-occurrence of vertebral defects, anal closure, cardiac defects, tracheoesophageal fistula, renal abnormalities, and limb abnormalities. To date, numerous reports exist regarding the surgical outcomes of CS associated with specific VACTERL association-type anomalies^3–5^. However, because CS has a heterogenetic and sporadic nature, causal and statistical approaches for determining the definite etiology and incidence rate are difficult to implement. Therefore, demographic studies on CS with statistical data are scarce.

The purpose of the present study is to investigate the incidence rate of CS among the age-matched normal population in Korea, as well as the features of CS treatment, by utilizing the National Health Insurance Service and Health Insurance Review and Assessment service (NHIS-HIRA) database.

## Materials and methods

### Data collection

The National Health Insurance (NHI) in the Republic of Korea is a public medical insurance system, covering the entire Korean population. All medical institutions, including private clinics, general hospitals attached to medical colleges, and higher-level hospitals, require NHI services to pay for medical expenses. After the HIRA reviews a claim, the NHI Service refunds the medical fee to the medical institution. As HIRA data are obtained from a billing form that is generated for each inpatient or outpatient visit to a medical facility, it is essential for the healthcare facility to enter the correct code associated with each diagnosis and prescription to receive the proper refund. Thus, this database, registered as the NHIS-HIRA database, contains detailed data on patient demographics, diagnoses, procedures, and prescription drugs, covering all citizens of South Korea.

**Figure 1:**
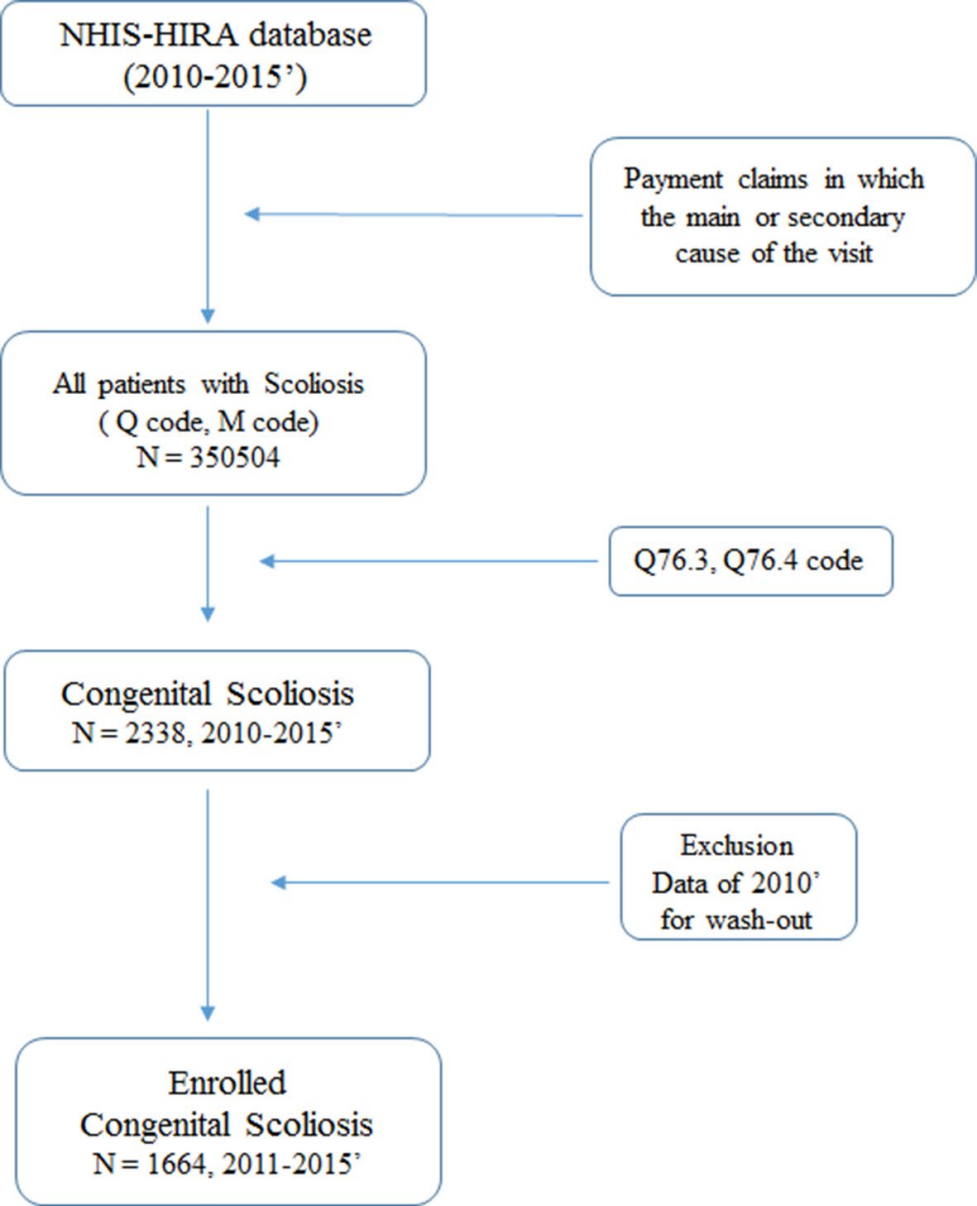
Diagram of the present nationwide scoliosis investigation of HIRA and NHI data. Cases of congenital scoliosis (defined as ICD-10th revision, Q76.4, Q76.3) were extracted from the NHIS-HIRA database (2010–2015). ICD-10th; International Classification of Diseases, 10th revision, HIRA; Health Insurance Review and Assessment Service, NHI; National Health Insurance, NHIS; National Health Insurance Service.

In the present study, we examined NHIS-HIRA data from January 1, 2010 to December 31, 2015. The data were filtered to determine the population with any type of scoliosis. We extracted only payment claims in which the main or secondary cause of the visit was scoliosis, as defined by the International Classification of Diseases, 10th revision (CS: Q76.4, Q76.3) (Fig. 1). All enrollees with these codes were confirmed by orthopedic surgeons or a pediatrician, the authors of this study. For the incidence estimates, the date of the earliest claim with the registration code for congenital scoliosis was defined as the index date. To eliminate any redundant potential pre-existing cases of congenital scoliosis, we established a 1-year wash-out period by excluding cases identified in the first 1 year (2010–2011). Therefore, patients first diagnosed with congenital scoliosis from 2011 to 2015 were included in the incidence estimates.

**Figure 2:**
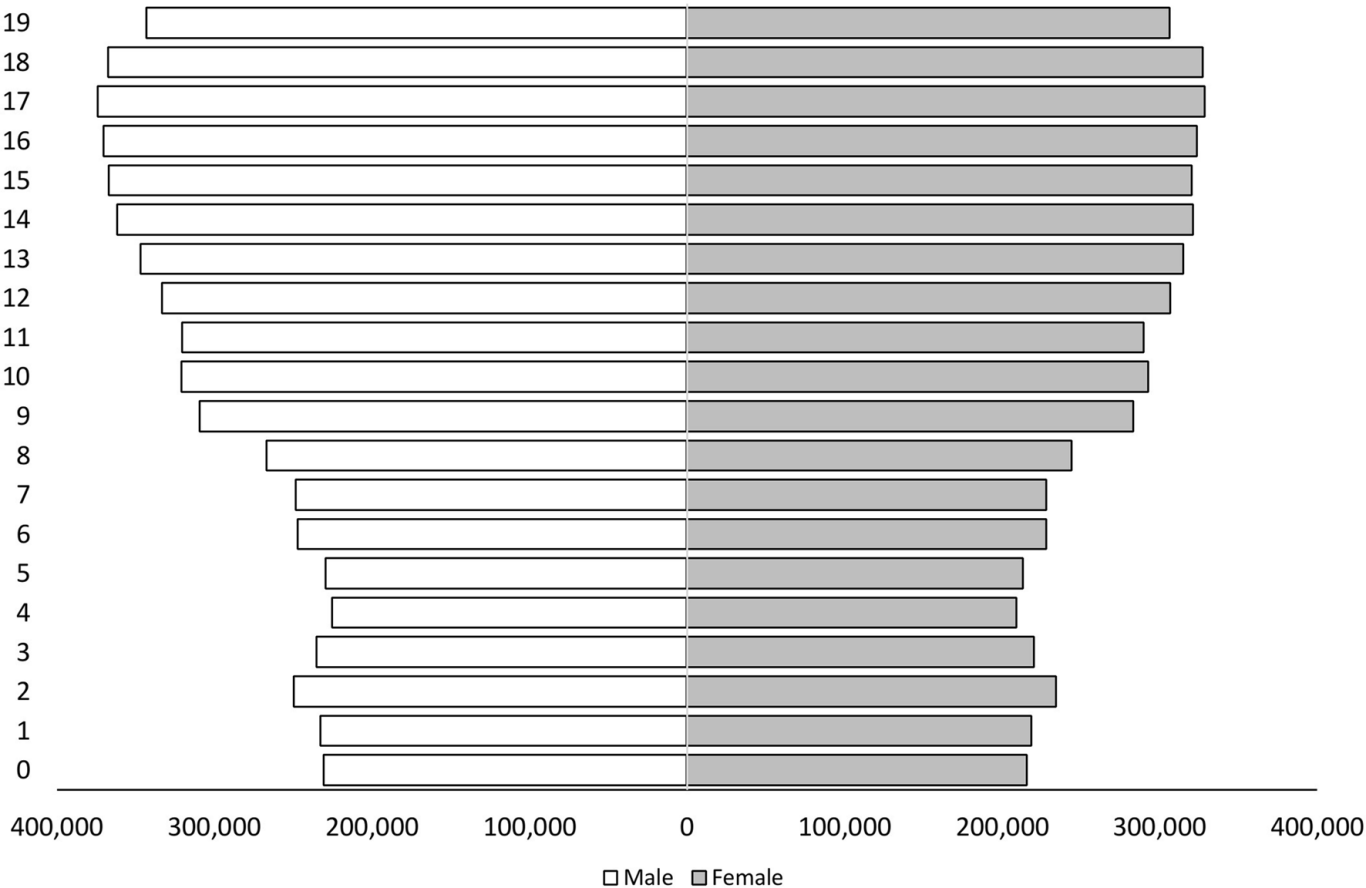
The population distribution by age group in 2010 in South Korea, based on Korean Statistical Information Service (KOSIS; http://kosis.kr) data. X-axis; total population according to sex, Y-axis; age in years (0–19 years).

Additionally, we used Korean Statistical Information Service (KOSIS; http://kosis.kr) data to determine the total population according to age during the study period, allowing the calculation of sex-specific and age-matched annual incidence rates (Fig. 2). This study was approved by our Institutional Review Board and Ethics Committee (Yonsei University Institutional Review Board and Ethics Committee: approval #2018-0062-001), which issued a waiver regarding the need for informed consent. All methods were performed in accordance with relevant guidelines and regulations.

### Calculation of the incidence rate of congenital scoliosis

The distribution of the population diagnosed with CS was determined overall and by sex and age (from 0 to 19 years of age). Data regarding sex and age at the initial diagnosis of CS (the time at which the code for CS was first registered in the NHIS-HIRA database) were collected and, using 1 and 5 year-age intervals, the sex- and age-specific incidence rates were calculated using the following formula:

$${\text{Number}}\,{\text{of}}\,{\text{diagnosed}}\,{\text{patients}}/{\text{number}}\,{\text{of}}\,{\text{individuals}}\,{\text{in}}\,{\text{the}}\,{\text{age - matched}}\,{\text{normal}}\,{\text{population}}$$

Incidence rates are reported in terms of the number per 100,000 persons in the age- and sex-matched normal population during a given period. A chi-square test was used to compare and analyze the average of the incidence rate per 100,000 between the period of 2nd growth spurt (male: 12 ~ 16 years, female: 10 ~ 14 years) and the period except 1st and 2nd growth spurt (male: 1 ~ 11 and 17 ~ 19 years, female: 1 ~ 9 and 15 ~ 19 years). The *p* value < 0.05 was considered statistically significant. 95% confidence intervals of each incidence rates are also presented.

### Proportion of patients who underwent surgical treatment for congenital scoliosis

We evaluated the proportion of patients who underwent surgical treatment (N0444, N0445 for anterior surgery; N0446, N0447 for posterior surgery) between 2011 and 2015 among those initially registered and followed-up in the NHIS-HIRA database during the study period. The overall proportion of surgical treatment and the proportion of anterior and posterior surgical treatment were evaluated.

## Results

### Incidence rate of congenital scoliosis

**Figure 3:**
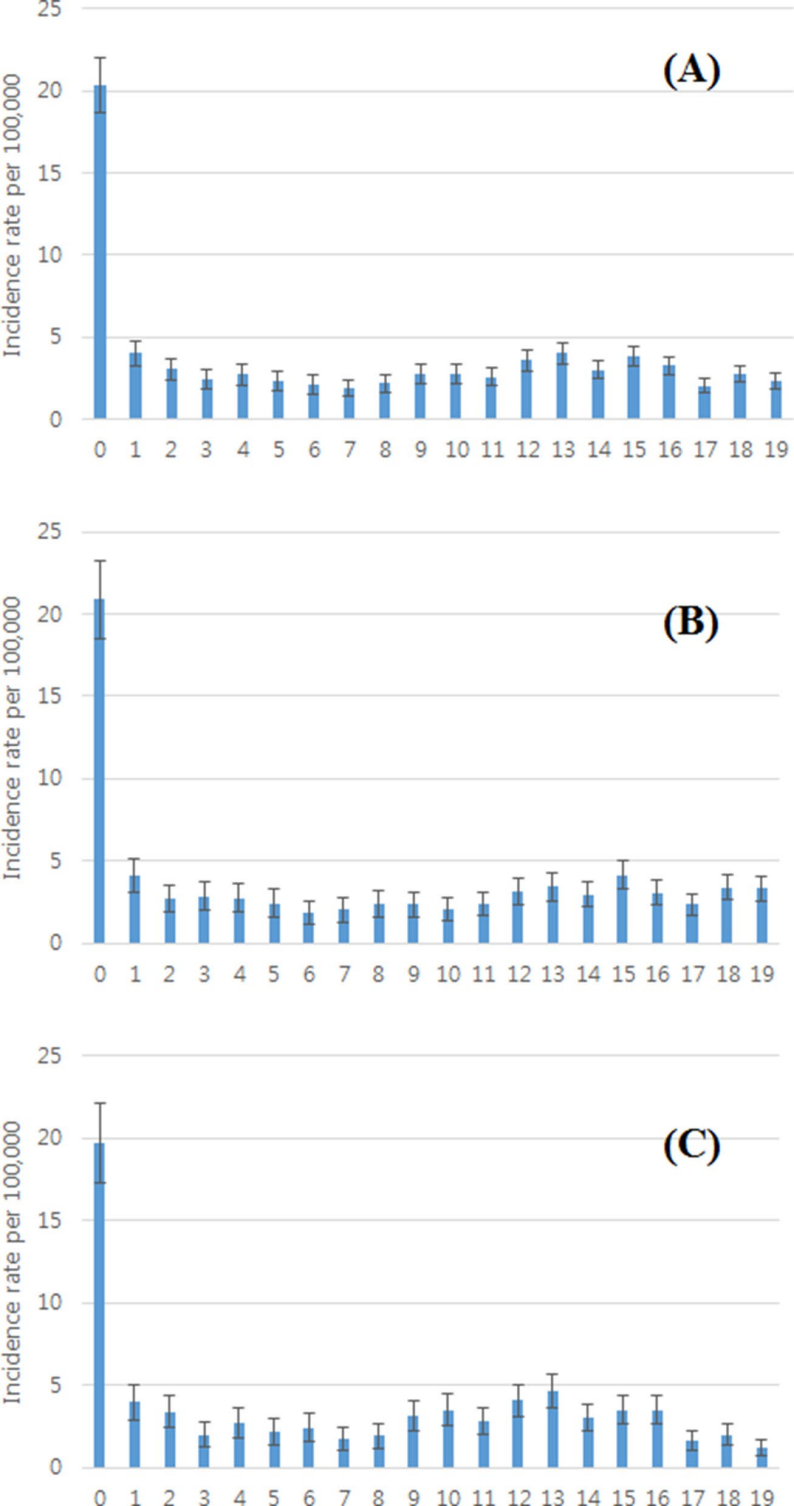
Sex-specific incidence rate of congenital scoliosis based on age (0–19 years), from 2011 to 2015: (**A**) Overall (female + males), (**B**) Males, and (**C**) Females. X-axis; age in years, Y-axis; incidence rate per 100,000. 95% confidence intervals are also indicated.

The total number of patients who were initially diagnosed with CS between 2011 and 2015 was 1664 (895 males and 769 females). The overall average incidence rate of CS over the 5-year study period was 3.08 per 100,000 persons. Stratified by sex, the average incidence rate of CS over the 5-year period was 2.99 per 100,000 persons in females and 3.18 per 100,000 persons in males. Among the 1664 patients with CS, 905 (54.4%) were diagnosed during the years in which growth spurts occur (age: 0 year and 12–16 years for males, 0 year and 10–14 years for females). Furthermore, 234 males and 211 females (26.1% and 27.4%) were diagnosed with CS during the second set of growth spurt years (in adolescence) (Table 1, Fig. 3).

**Table 1:** Individual annual and 5-years average incidence rate of congenital scoliosis from 2011 to 2015.

Year	Total population (0 ~ 19 years old)			Congenital scoliosis patients (0 ~ 19 years old)			Incidence rate per 100,000		
	Overall	Male	Female	Overall	Male	Female	Overall	Male	Female
2011	11,239,996	5,881,170	5,358,826	441	237	204	3.92	4.03	3.81
2012	11,012,807	5,753,390	5,259,417	365	203	162	3.31	3.53	3.08
2013	10,765,287	5,611,677	5,153,610	320	161	159	2.97	2.87	3.09
2014	10,503,422	5,461,478	5,041,944	248	148	100	2.36	2.71	1.98
2015	10,252,151	5,317,005	4,935,146	290	146	144	2.83	2.75	2.92
5-years average							3.08	3.18	2.99

Stratified by age, the overall average incidence rate of CS over the 5-year period ranged from 1.5 to 20.1 per 100,000 persons. The highest incidence rate was observed at 0 years of age, and followed a parabolic shape, pointing downward, with a second incidence rate peak at 12–16 years of age. Further stratified by sex, the average incidence rate of CS over the 5-year period ranged from 1.2 to 20.4 per 100,000 persons in males and from 1.1 to 19.8 per 100,000 persons in females.

**Figure 4:**
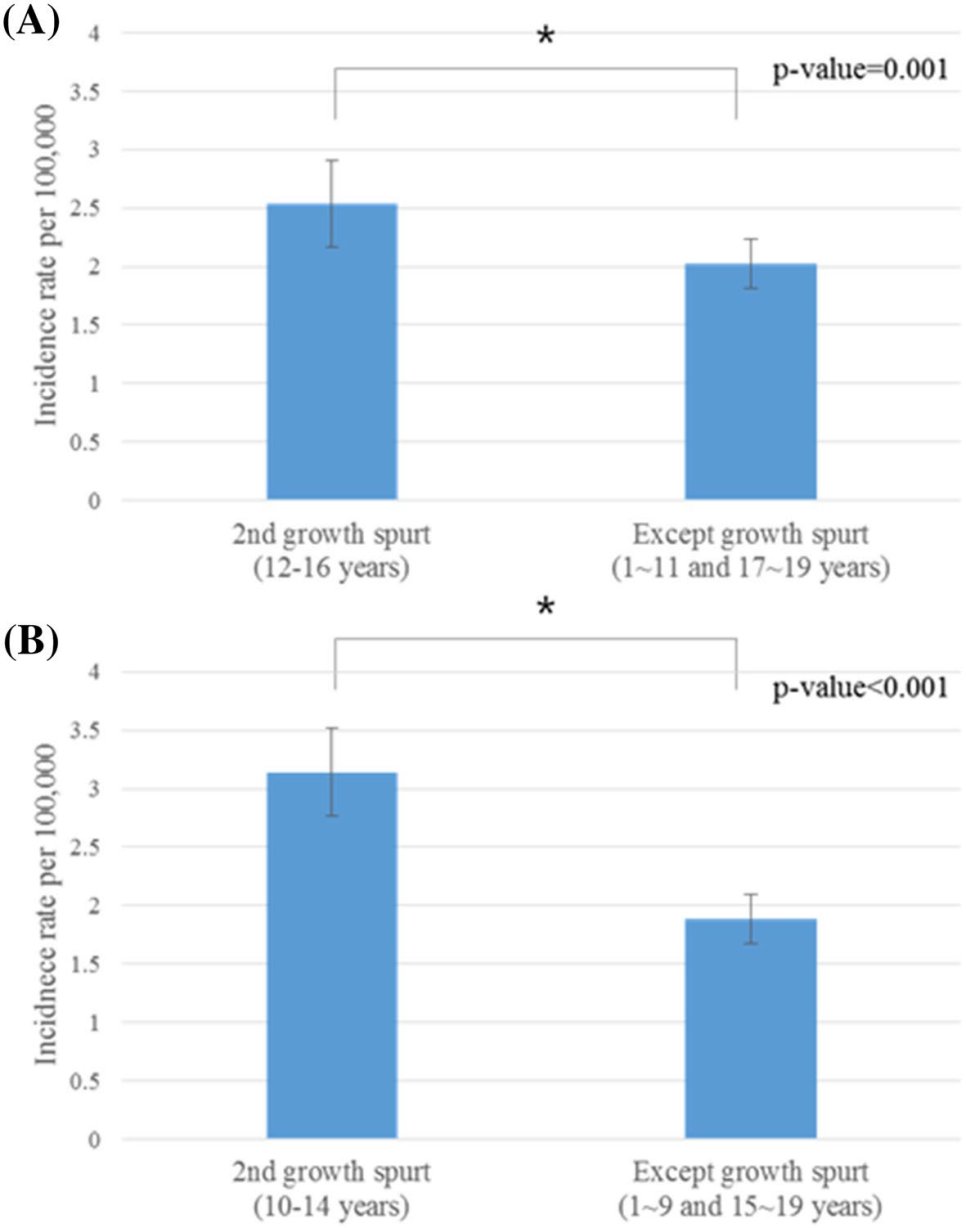
Comparison of Age-specific incidence rate between 2nd growth spurt period (Male: 12–16 years, Female: 10–14 years) and except growth spurt period, from 2011 to 2015: (**A**) Males, and (**B**) Females. X-axis; age in years, Y-axis; incidence rate per 100,000. 95% confidence intervals are also indicated. * means that the two groups are statistically significantly different.

As a result of comparing the overall data including incidence rate per 100,000 people between 2nd growth spurt period (Male: 12–16 years, Female: 10–14 years) and except growth spurt period, from 2011 to 2015, there were significant differences as *p* = 0.001 for male and *p* < 0.001 for female (Fig. 4).

For males, the highest incidence rate was observed at 0 years of age (20.4 per 100,000 persons), followed by that at 15 years of age (3.65 per 100,000 persons). For females, the highest incidence rate was observed at 0 years of age (19.8 per 100,000 persons), followed by that at 13 years of age (4.0 per 100,000 persons). Thus, the highest incidence rate occurred at 0 years of age in both males and females, but the second highest incidence rate occurred approximately 2 years later in males than in females (Tables 2, 3, Fig. 3).

**Table 2:** Sex-specific and age-specific annual incidence rate of congenital scoliosis for 5 years (2011–2015).

Year	2011			2012			2013			2014			2015		
	Overall	Male	Female	Overall	Male	Female	Overall	Male	Female	Overall	Male	Female	Overall	Male	Female
**Age**															
0	18.64	20.93	16.22	15.82	18.44	13.04	20.01	14.68	25.62	21.52	25.45	17.38	24.45	22.52	26.49
1	4.50	4.37	4.65	2.91	4.45	1.29	4.66	4.12	5.24	2.37	2.52	2.21	3.68	3.14	4.25
2	2.88	1.72	4.12	2.03	2.19	1.86	2.50	4.05	0.86	1.91	2.47	1.31	2.38	1.26	3.55
3	2.28	2.81	1.71	1.78	1.72	1.83	2.03	3.06	0.93	0.83	0.00	1.72	1.70	2.48	0.87
4	3.30	3.41	3.18	2.07	1.61	2.56	1.34	0.86	1.84	1.58	2.63	0.47	1.88	1.22	2.57
5	3.00	4.45	1.44	1.32	1.28	1.37	1.66	2.01	1.29	1.78	0.86	2.76	0.90	0.88	0.93
6	2.04	1.75	2.35	1.85	1.34	2.40	0.66	0.86	0.46	1.46	1.21	1.72	1.79	0.87	2.77
7	2.74	3.65	1.76	2.49	3.06	1.88	1.39	1.34	1.44	0.44	0.43	0.46	1.04	0.81	1.29
8	0.42	0.40	0.44	2.74	2.84	2.63	1.59	1.75	1.41	1.16	0.89	1.44	2.22	2.58	1.83
9	1.57	0.75	2.46	2.52	2.02	3.07	1.90	1.22	2.64	1.59	2.19	0.94	1.86	2.24	1.45
10	4.05	2.91	5.30	1.96	1.13	2.87	1.68	1.62	1.76	1.27	1.22	1.32	2.28	1.76	2.83
11	2.61	2.18	3.07	3.38	2.92	3.89	4.12	3.76	4.51	1.47	2.43	0.44	0.64	0.41	0.88
12	5.57	4.68	6.55	4.06	3.42	4.77	1.86	1.29	2.47	1.96	2.63	1.23	1.89	1.62	2.20
13	6.08	6.29	5.85	4.26	4.06	4.48	2.76	2.80	2.72	1.86	1.29	2.47	3.33	2.25	4.51
14	4.08	3.47	4.76	3.43	3.60	3.25	2.30	2.50	2.07	1.14	0.93	1.36	1.01	1.29	0.71
15	3.22	3.88	2.49	4.39	5.21	3.49	3.43	4.50	2.27	1.64	1.57	1.72	2.11	3.11	1.02
16	3.65	3.01	4.38	2.79	1.94	3.74	2.27	1.45	3.17	2.18	3.30	0.97	1.81	1.57	2.07
17	1.73	1.89	1.54	1.31	1.64	0.94	1.90	1.38	2.49	1.36	1.74	0.95	1.24	1.79	0.65
18	3.68	4.53	2.73	2.87	3.77	1.84	1.74	3.00	0.31	1.17	1.38	0.93	1.96	3.18	0.63
19	3.14	4.59	1.51	2.67	4.23	0.90	1.71	2.41	0.92	1.74	2.71	0.62	1.88	2.19	1.53

*Annual incidences are represented in terms of the number per 100,000 persons.

*Korean Statistical Information Service (KOSIS; http://kosis.kr) data was used to determine the total population according to age during the study period, allowing the calculation of sex-specific and age-matched annual incidences.

**Table 3:** Sex-specific and age-specific average incidence rate of congenital scoliosis for 5 years (2011–2015).

Year	Overall			Male			Female		
	95% CI Lower limit	Average	95% CI Upper limit	95% CI Lower limit	Average	95% CI Upper limit	95% CI Lower limit	Average	95% CI Upper limit
**Age**									
0	17.27	20.09	22.91	16.82	20.40	23.98	14.51	19.75	24.99
1	2.76	3.63	4.49	2.98	3.72	4.46	2.04	3.53	5.01
2	2.00	2.34	2.68	1.41	2.34	3.27	1.09	2.34	3.59
3	1.24	1.72	2.20	0.93	2.01	3.10	1.00	1.41	1.82
4	1.36	2.03	2.70	1.02	1.95	2.87	1.21	2.12	3.04
5	1.05	1.73	2.42	0.58	1.90	3.21	0.94	1.56	2.17
6	1.08	1.56	2.03	0.88	1.20	1.53	1.14	1.94	2.73
7	0.77	1.62	2.47	0.61	1.86	3.10	0.87	1.36	1.86
8	0.83	1.63	2.42	0.77	1.69	2.62	0.86	1.55	2.25
9	1.55	1.89	2.23	1.10	1.68	2.27	1.34	2.11	2.89
10	1.31	2.25	3.19	1.10	1.73	2.35	1.46	2.82	4.17
11	1.21	2.44	3.68	1.25	2.34	3.42	0.97	2.56	4.15
12	1.59	3.07	4.54	1.52	2.73	3.94	1.54	3.44	5.34
13	2.24	3.66	5.07	1.65	3.34	5.03	2.77	4.01	5.24
14	1.20	2.39	3.58	1.29	2.36	3.43	1.02	2.43	3.84
15	2.00	2.96	3.92	2.43	3.65	4.88	1.39	2.20	3.00
16	1.92	2.54	3.16	1.51	2.25	2.99	1.68	2.87	4.05
17	1.26	1.51	1.76	1.52	1.69	1.86	0.67	1.31	1.95
18	1.41	2.28	3.16	2.15	3.17	4.19	0.42	1.29	2.15
19	1.66	2.23	2.79	2.26	3.23	4.19	0.74	1.10	1.45

*Annual incidences are represented in terms of the number per 100,000 persons.

*Korean Statistical Information Service (KOSIS; http://kosis.kr) data was used to determine the total population according to age during the study period, allowing the calculation of sex-specific and age-matched annual incidences.

*95% CI indicates 95% confidence interval.

### Proportion of patients who underwent surgical treatment

A total of 92 (5.5%) patients diagnosed with CS during the study period underwent surgery within 5 years after the initial diagnosis. Among these, 14 patients were diagnosed at 15–19 years of age (9 males and 5 females), 52 patients (56.5%) were diagnosed at 10–14 years of age (25 males and 27 females), 15 were diagnosed at 5–9 years of age (5 males and 10 females); and 11 patients (12.0%) were diagnosed at 0–4 years of age (4 males and 7 females). In total, 57 of the surgical cases were male and 75 were female; the difference between the sexes failed to reach significance (*p* = 0.163).

**Figure 5:**
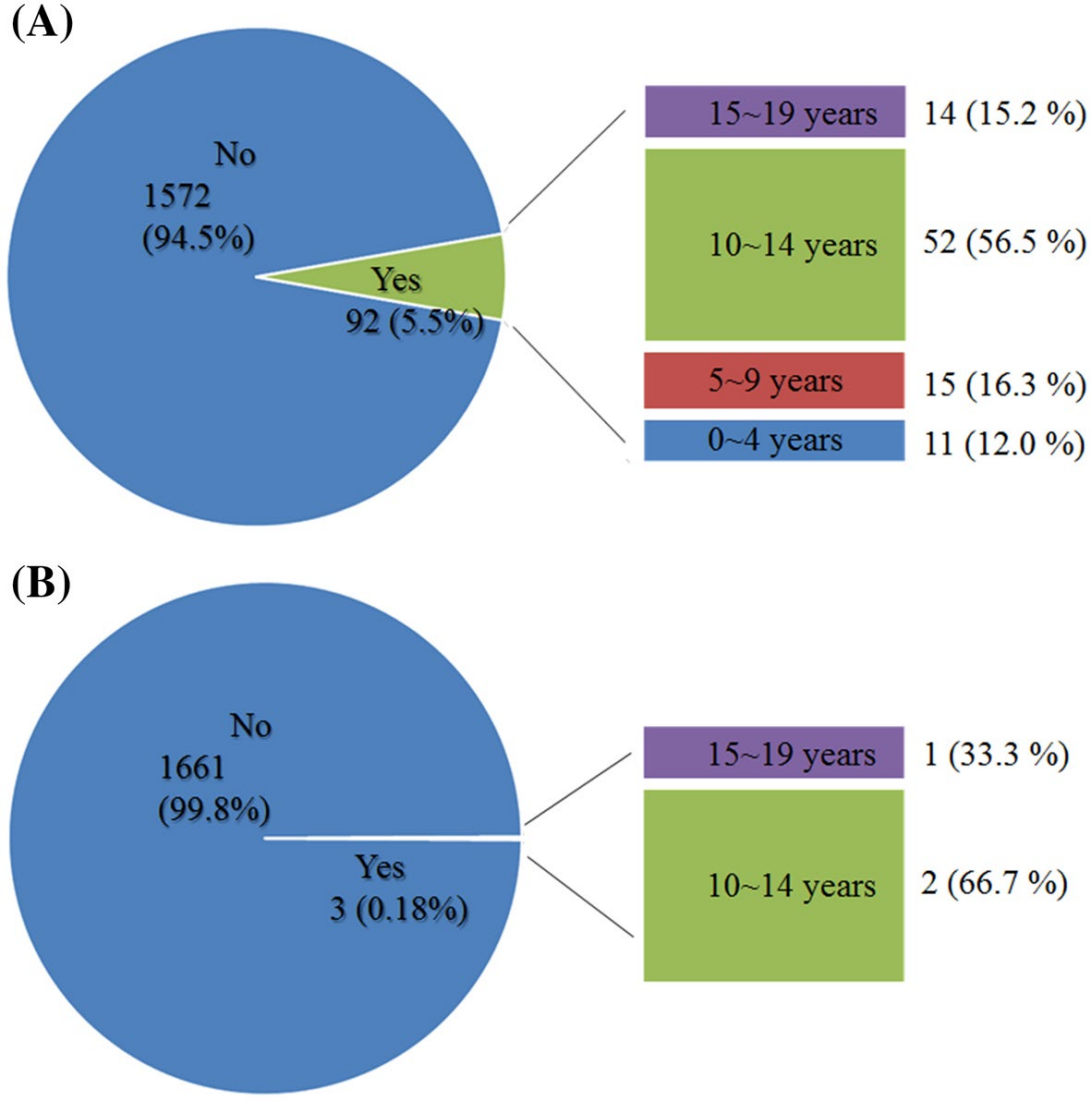
Diagrams showing the proportion of patients with CS who underwent surgical treatment: (**A**) Posterior surgical treatment, and (**B**) Anterior surgical treatment. Data were extracted through the following procedure codes (N0444, N0445 for anterior surgery; N0446, N0447 for posterior surgery), from 2011 to 2015. Patients corresponding to ‘Yes’ are separately stratified according to age, and the patterns of patients who received surgical treatment are further described according to age group. N (n%): N, Number of age-stratified patients among all patients undergoing surgery; n, Percentage of all age-stratified patients who underwent surgery.

Anterior surgery (N0444, N0445) was performed in only 3 patients (0.18% of all patients with CS and 3.26% of surgically treated patients with CS). In contrast, posterior surgery (N0446, N0447) was performed in 92 patients (5.5% of all patients with CS). 11 patients (12.0%) diagnosed at 0–4 years of age, 15 (16.3%) patients diagnosed at 5–9 years of age, 52 (56.5%) patients diagnosed at 10–14 years of age, and 14 (15.2%) patients diagnosed at 15–19 years of age underwent posterior surgery. Thus, most surgical interventions comprised posterior surgery, with the highest prevalence (56.5%) in patients diagnosed with CS at 10–14 years of age (Fig. 5).

## Discussion

This study is the first population-based epidemiological study on CS in the Republic of Korea, which has a population of approximately 50 million. Due to its rarity, little is known about the incidence rate of CS. Previous studies have reported that congenital spinal malformations occur at a frequency of 0.01–1.0 per 1000 live births, accounting for about 8.9% of all structural scoliosis cases, and are 2.5 times more prevalent in females^6–8^. However, in the present study, the 5-year average incidence rate was 3.08 per 100,000 persons, with a slightly higher rate in male than in females. Recently, von Heideken et al. reported an overall annual incidence rate of CS of 0.7 per 100,000 persons using a Swedish nationwide sampling^8^. However, as this previous nationwide study focused on patients who underwent surgical treatment in Sweden, it is not surprising that the incidence was much lower than that in the present study, which focused on those diagnosed with CS among the total population.

Additionally, Passias et al., using data from the Healthcare Cost and Utilization Project's Kids Inpatient Database (KID) in the United States, reported that the incidences of the congenital spinal abnormalities, hemivertebra and the absence of vertebra, were 9.1 and 1.2 per 100,000 persons, respectively, which differ from those in the present report^9^. The study by Passias et al. focused on VACTERL syndrome associated with congenital spinal abnormalities; thus, the difference in study results might be related to the use of different codes for reimbursement in the International Classification of Disease, Ninth Revision, and Clinical Modification (ICD-9-CM) formats.

The shape and extent of spinal deformities in patients with congenital spinal malformations vary greatly^6,10^. Although many patients do not experience spinal deformities or imbalance problems during their lifetime, early deformities can result in problems, such as cardiopulmonary decline and spinal cord compression^11,12^. Considering the diversity in the natural course of CS, numerous studies have focused on the prediction of the natural course in patients with CS^2,13,14^. However, the prediction accuracy has not yet reached a satisfactory level, and trends in the initial patient incidence rate between the ages of 0–19 years have not been considered.

Moreover, CS is a congenital anomaly of the spine associated with abnormalities inside and outside of the spine^10,15–17^. VACTERL syndrome usually involves 3 or more previous VACTERL association-type anomalies. Therefore, it is expected that the highest incidence rate of CS would be observed in newborns with symptoms related to VACTERL association-type abnormalities. Furthermore, CS is often clinically significant at the time of the first growth spurt, as the patient's growth pattern becomes noticeably below normal threshold values^2,10^. Subsequently, the scoliotic angle progresses during the second growth spurt period, which is associated with longitudinal and volumetric growth, potentially leading to an increase in the diagnosis of CS^14^.

The age- and sex-specific incidence rates showed a characteristic downward-pointing parabolic shape in the present study, with two peaks at 0 years and 12–16 years of age. Previous studies have reported that spinal deformities, usually caused by congenital vertebral malformations, tend to progress rapidly during infancy and pre-adolescent growth spurts, and tend to be relatively slow during mid-childhood (5–10 years of age)^2,8,10^. This pattern in progression is thought to exist because spinal deformities result from an imbalance in spinal length growth (due to an unbalanced distribution of the superior and inferior endplates), stemming from congenital malformations. This conception is consistent with the results of the present study. According to a report of 250 congenital scoliosis patients conducted by McMaster et al., the bimodal pattern showed the highest incidence rate in the 1st growth spurt period, followed by the second highest incidence rate in the 2nd growth spurt period. Showed. Although this study was a total survey of approximately 50 million people, the results were similar to the above. Therefore, it can have a clinical meaning to recognize that congenital scoliosis can be diagnosed even in adolescence.

Additionally, in the present study, the second peak in the incidence rate of CS occurred about 2 years earlier in females than in males, consistent with the fact that the second growth spurt occurs approximately 2 years earlier in females than in males^18,19^. This difference in growth spurt tendency may also influence the number of times that patients with CS visit a medical facility owing to symptoms, such as those associated with VACTERL anomalies, CS-related symptoms, and pain due to scoliosis angle progression.

The treatment of the spinal deformities due to congenital vertebral malformations remains the domain with the most room for development. Ideally, spinal deformities and other congenital abnormalities should be treated early, and the natural course of the spinal deformity should be predicted in advance to avoid spinal deformity advancement and secondary deformities. However, unlike in adolescent idiopathic scoliosis, a quantitative Cobb angle for the recommendation of surgical treatment has not been established^20–22^. Therefore, it is essential to closely observe the progression of the lesion, as this is the most important factor determining the need for surgical treatment. Information regarding the type of surgical treatment performed, and the age at which it is performed, will be helpful in understanding the appropriate treatment of the lesion. The age at the time of surgery varies depending on the surgical conditions and settings. Surgery performed at an appropriate age, when there is less impairment in pulmonary function, can lead to better surgical outcomes^8,20^.

The present study provides new information regarding the latest trends in the surgical treatment of CS. The surgery rate was the highest (56.5% of all surgical cases) among the patients who were diagnosed at an age of 10–14 years. Furthermore, the noticeable difference in frequencies of anterior and posterior surgeries is a reflection of the advancement in methods that are only used in posterior surgery for the treatment of CS^4,23^.

This study has several limitations. First, the most commonly used classification system for CS is the modified method of McMaster and Ohtsuka^2^. This system classifies CS into a formation defect, segmentation defect, or complex malformation, according to the mechanism of development. However, detailed incidence rates of CS subtypes, as well the most vulnerable type of surgical treatment implementation, could not be determined in the present study.

Second, to determine the proportion of patients with CS who underwent surgical treatment, we extracted claims using the code for anterior surgery (N0444, N0445) or posterior surgery (N0446, N0447) from the NHIS-HIRA database. CS is closely related to the development of the spinal mesoderm, and it is known that 61% of patients with CS have congenital malformations of other types occurring in the mesoderm. As the code for surgical treatment owing to the expression of VACTERL-association syndromes was an exclusion criterion, the data of patients with CS who underwent surgical treatment for extraspinal anomalies were not included in the surgical treatment analysis. In addition, growing rod instrumentation and vertical expandable prosthetic titanium ribs (VEPTR) have emerged as treatments for patients with spinal deformities aged over 5 years, while maintaining growth, and surgical treatments to inhibit progression have been developed^3,24,25^. Currently, there are no surgical codes associated with VEPTR surgeries in the NHIS-HIRA system, and we cannot assume that VEPTR surgeries were included in the posterior surgery code.

Third, since the modified classification of CS according to McMaster and Ohtsuka cannot be classified according to the NHIS-HIRA database, there is a risk of missing congenital malformation of spine that did not progress at the time of diagnosis. For this, Q76.3, which is a non-billable code and codes for the congenital malformation of spine, in which the scoliotic angle did not progress at the time of diagnosis, was included. As a result, the proposed result may be over-estimation or underestimated, resulting in a limitation that must be interpreted carefully.

## Conclusion

In conclusion, the present Korean epidemiological study on CS, the first of its kind, estimated the average incidence rate of CS over a 5-year period (2011 to 2015) as 3.08 per 100,000 persons. The age-specific CS incidence rate was highest at 0 years of age, and showed a downward-pointing parabolic shape, with a second peak during adolescence, at the time of the second growth spurt. Additionally, only 5.5% of patients with CS underwent surgery within 5 years of the initial diagnosis.
